# Novel Liposomal Formulation of Baicalein for the Treatment of Pancreatic Ductal Adenocarcinoma: Design, Characterization, and Evaluation

**DOI:** 10.3390/pharmaceutics15010179

**Published:** 2023-01-04

**Authors:** Adam Markowski, Magdalena Zaremba-Czogalla, Anna Jaromin, Ewa Olczak, Adrianna Zygmunt, Haniyeh Etezadi, Ben J. Boyd, Jerzy Gubernator

**Affiliations:** 1 Department of Lipids and Liposomes, Faculty of Biotechnology, University of Wrocław, Joliot-Curie 14a, 50-383 Wrocław, Poland; 2 Department of Pharmacy, University of Copenhagen, Universitetsparken 2, 2100 Copenhagen, Denmark; 3 Drug Delivery, Disposition and Dynamics, Monash Institute of Pharmaceutical Sciences, Monash University (Parkville Campus), 381 Royal Parade, Parkville, VIC 3052, Australia

**Keywords:** liposomes, baicalein, pancreatic cancer, flavonoids, hemolysis, nanotechnology, nanomedicine, natural compounds

## Abstract

Pancreatic cancer (PC) is one of the deadliest cancers so there is an urgent need to develop new drugs and therapies to treat it. Liposome-based formulations of naturally-derived bioactive compounds are promising anticancer candidates due to their potential for passive accumulation in tumor tissues, protection against payload degradation, and prevention of non-specific toxicity. We chose the naturally-derived flavonoid baicalein (BAI) due to its promising effect against pancreatic ductal adenocarcinoma (PDAC) and encapsulated it into a liposomal bilayer using the passive loading method, with an almost 90% efficiency. We performed a morphological and stability analysis of the obtained BAI liposomal formulation and evaluated its activity on two-dimensional and three-dimensional pancreatic cell models. As the result, we obtained a stable BAI-encapsulated liposomal suspension with a size of 100.9 nm ± 2.7 and homogeneity PDI = 0.124 ± 0.02, suitable for intravenous administration. Furthermore, this formulation showed high cytotoxic activity towards AsPC-1 and BxPC-3 PDAC cell lines (IC_50_ values ranging from 21 ± 3.6 µM to 27.6 ± 4.1 µM), with limited toxicity towards normal NHDF cells and a lack of hemolytic activity. Based on these results, this new BAI liposomal formulation is an excellent candidate for potential anti-PDAC therapy.

## 1. Introduction

According to the World Health Organization, in 2020, almost 10 million people died from cancer diseases, with lung, stomach, colon, and liver cancer being the most deadly. However, according to the same data, pancreatic cancer was responsible for almost half a million deaths, which brings this type of cancer to sixth place among cancer-related deaths in 2020 [1]. It is predicted that by 2025, pancreatic cancer will take third place in cancer-related deaths in the EU (European Union) [2]. Of all diagnosed pancreatic cancer cases, over 90% are pancreatic ductal adenocarcinoma [3]. A PDAC is characterized by high desmoplasia, increased chemoresistance, early and rapid metastasis, and prolonged and inconspicuous disease progression. Most of the diagnoses are made at late stages of cancer development when successful surgical resection—the only truly effective way to cure patients with PDAC—is impossible. Usually, most diagnoses result in death in the next six months [4,5].

Currently, most anti-PDAC therapies are based on gemcitabine, the most frequently used chemotherapeutic. However, treatment with gemcitabine alone results in a relatively poor response from patients, only improving life quality for the final months. The purpose of using drugs such as gemcitabine is only to improve the quality of life for PDAC patients, not to treat the disease in any way [6]. Other strategies are based on therapies such as a combination of gemcitabine with cisplatin or FOLFIRINOX, which is a mixture of folinic acid, 5-fluorouracil, irinotecan, and oxaliplatin. The outcome of using FOLFIRINOX is a prolonged lifespan of up to 11 months, which doubles the results from using a single gemcitabine treatment. Unfortunately, only a small fraction of patients qualify for this treatment due to the many side effects of FOLFIRINOX usage [7]. Therefore, there is no effective drug on the market which can effectively treat PDAC patients.

As mentioned above, there are several major factors that determine the high mortality in PDAC patients. The first is high desmoplasia correlated with cancer cells. Most of the cancer cell mass is composed of cancer-associated fibroblasts (CAF), pancreatic stellate cells (PSC), and a vast spectrum of the products of these cells: fibronectins, lamins, ezrins, and hyaluronic acid, which are components of the extracellular matrix (ECM) of the PDAC. This very dense ECM creates a physical and chemical wall, preventing molecules from penetrating it and enacting their biological effect against cancer cells [8,9]. Another aspect is the low immunogenicity of cancer because of a decreased number of antigens present on the cell surface. In addition, pancreatic cells present a large number of immunosuppressive agents, which, along with the poor penetration of killer immune cells, leads to the immune escape of PDAC with extremely difficult immunotherapy and invisibility from the immune system during progression. The KRAS mutation is primarily responsible for this immune escape, which can be seen as the primary mutation responsible for all subsequent PDAC-related carcinogenic phenomena. It is responsible for both the promotion of healthy pancreatic cell lesions to tumor-transformed PanINs (Pancreatic Intraepithelial Neoplasias), the cross-talk between tumor cells and fibroblasts resulting in the production of huge amounts of extracellular matrix, and the silencing of the immune system or the promotion of inflammation. This is a mutation that tightly regulates phenomena in the PDAC microenvironment [10,11]. Moreover, PDAC has a tendency for metastasis at the earliest stages of the disease, with most ascites located in the lungs and liver [12].

One of the most promising weapons to fight against PDAC is the use of nanomedicine in the form of liposomes, micelles, or nanoparticles. The enhanced production of pro- and anti-angiogenic factors, such as angiostatin and endostatin by PDAC, leads to structural and functional abnormalities of cancer vessels. These abnormalities lead to the phenomenon known as enhanced permeability and retention (EPR), where drug nanocarriers can leak from the structural defects of blood vessels into the tumor mass [13,14,15]. This phenomenon of nanocarrier accumulation is observed, for example, in the chemotherapeutic Doxil, where using liposomal doxorubicin significantly enhances the pharmacokinetics of the doxorubicin and its therapeutic potential [16]. Liposomes are spherical entities composed of phospholipids with additives such as cholesterol, sphingolipids, and detergents. They can vary in size, ranging from 20 nm up to 1000 nm, lipid composition, surface potential, and surface modifications [17,18]. Liposomes are a platform for the encapsulation and delivery of various water-soluble and water-insoluble drugs such as doxorubicin, epirubicin, cytarabine, amphotericin B, and a variety of naturally derived polyphenols and terpenoids including paclitaxel, curcumin, quercetin, and berberine [19,20,21,22,23,24,25,26,27]. The ability to manipulate the lipid composition makes it possible to encapsulate and achieve the desired biological effect from potentially valuable but poorly bioavailable compounds because of their hydrophobic nature [28]. Liposome surface modification in the form of antibodies, peptides, and PEG (Polyethylen glycol) addition can further enhance their anticancer potential [29,30].

Flavonoids are a large group of compounds synthesized in plants as bioactive secondary metabolites which are responsible for their color, flavor, and some pharmacological activities. Their main sources are fruits, vegetables, and other plant products including black and green tea, red wine, and various herbs [31]. It is important to maintain a diet rich in flavonoids due to their pro-health properties. Flavonoids have been reported as excellent antioxidants, fat intake reducers, and glucose absorption inhibitors, and promote the maintenance of the colon microflora [32,33,34,35]. In addition, flavonoids possess many valuable anticancer properties: they are excellent reactive oxygen species (ROS) scavengers and participate in cell cycle arrest. They are also apoptosis and autophagy activators and suppressors of cancer cell proliferation and invasiveness [36]. Several flavonoids have been tested against PDAC, including quercetin, kaempferol, apigenin, luteolin, and specific cannabis-originated flavonoids, such as FBL-03G, as well as baicalein, the flavonoid which is the focus of this work [37,38,39,40,41,42].

Baicalein (BAI) is one of the major bioactive constituents of *Scutellaria baicalensis,* isolated from the roots of this plant together with other flavonoids such as baicalin, wogonin, and norwogonin [43]. BAI exerts multiple pharmacologic effects including anti-inflammatory, antioxidant, and antiviral action as well as cardiovascular protection [44]. However, of prime importance is the finding that BAI demonstrates a high anticancer potential. The main molecular mechanisms of the anti-tumor effects of baicalein include cyclin-dependent kinase (CDK) dependent cell cycle inhibition, ROS scavenging, attenuation of MAPK/Akt/TOR pathways, as well as apoptosis induction through caspase-9/-3 activation and inhibition of cancer metastasis and invasiveness by the downregulation of MMP-2/-3 metalloproteinases. Baicalein is reported as an inhibitor of various cancers including lung, prostate, ovarian, colorectal, breast, and—what is most important in the context of this work—pancreatic cancer, where BAI exerts anti-PDAC activities such as the inhibition of the 5-LOX and 12-LOX enzymes, which results in reduced cellular growth. Furthermore, BAI is responsible for the cytochrome c-dependent apoptosis of PDAC cells as well as increasing the ratio between Bax/Bcl-2 proteins [45]. BAI also downregulates the expression of the Mcl-1 protein and its mRNA which are responsible for the chemoresistance of pancreatic cancer cells [46]. Unfortunately, besides its many beneficial anticancer properties, BAI is characterized by very low bioavailability. This is due to its low water solubility, degradation in the digestive system, the strong metabolism of the compound, and its rapid clearance from systemic circulation. Therefore, a liposomal formulation of BAI is anticipated to achieve a proper therapeutic effect and plasma concentration as well as an increased concentration in cancer tissues by protecting the drug from degradation and uncontrolled metabolism [47,48,49].

This work focused on the preparation of a liposomal formulation of BAI (BAI-L) which was characterized morphologically; in addition, the entrapment efficiency and stability were determined and the compound retention in liposomes during incubation with FBS was assessed. The cytotoxicity of BAI-L towards two PDAC cell lines, primary BxPC-3 and ascites AsPC-1, was determined, as was its toxicity towards a normal human dermal fibroblast (NHDF) cell line and human erythrocytes. Additionally, the 3D-ATP generation in a more modern approach in a 3D spheroid culture was studied. The cellular uptake of BAI liposomes was assessed as well as their visualization using the cryo-TEM method. Lastly, the impact of BAI on DNA fragmentation was evaluated.

The present work has a chance to shed light on the methods of preparing liposomal preparations based on flavonoids, i.e., how to encapsulate them quickly and efficiently. Based on previous data on the relatively low-efficiency encapsulation of hydrophobic compounds of natural origin, this work can help answer questions and solve potential problems arising from working with this type of material. The production of flavonoid-based liposomal formulations is not an easy or trouble-free process, so we hope that this work will provide some novelty and help other scientific groups in their work.

## 2. Materials and Methods

### 2.1. Materials

1,2-Dimyristoyl-sn-glycero-3-phosphocholine (DMPC), N-(carbonyl-methoxypolyethylenglycol-2000)-1,2-distearoyl-sn-glycero-3-phosphoethanolamine, and Na-salt (DSPE-PEG 2000) were purchased from Lipoid, Ludwigshafen, Germany. Baicalein was purchased from Haoxuan Biotech, Xi’an, China. Chloroform, methanol, and tert-butanol were purchased from Stanlab, Lublin, Poland. Sodium chloride, dimethylsulfoxide (DMSO), and ethylenediaminetetraacetic acid (EDTA) were purchased from ChemPur, Piekary Śląskie, Poland. Sepharose 4B CL, thiazolyl blue tetrazolium bromide, 2-(4-amidinophenyl)-6-indolecarbamidine dihydrochloride, and 4′,6-diamidino-2-phenylindole dihydrochloride (DAPI) were purchased from Merck, Darmstadt Germany. The DiL stain (1,1ʹ-dioctadecyl-3,3,3’,3’-tetramethylindocarbocyanine perchlorate) was purchased from Thermo Fisher, Waltham, USA. The RPMI-1640 and MEM-Alpha cell culture media were purchased from Lonza, Basil, Switzerland. Fetal bovine serum (FBS), GlutaMAX (L-alanyl-L-glutamine dipeptide in 0.85% NaCl), and 100× antibiotic-antimycotic were purchased from BioWest, Nuaillé, France. Carbon films were purchased from Lacey Formvar/Cu grids, SPI Supplies, West Chester, PA, USA. Agarose was purchased from Roth, Karlsruhe, Germany. The CellTiter-Glo 3D Viability Assay and RealTime-Glo Annexin V Apoptosis and Necrosis Assay were purchased from Promega, Madison, WI, USA.

### 2.2. Liposome Preparation

**Figure 1:**
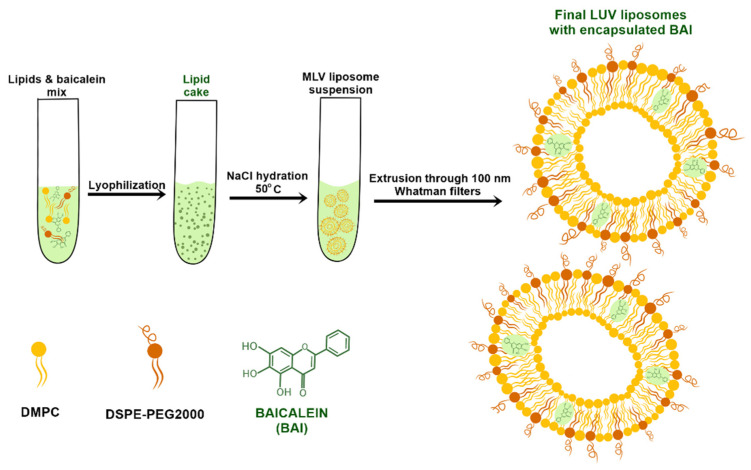
Diagram showing the preparation of liposomal baicalein.

Liposomes were prepared using the lipid cake hydration method. Briefly, lipids were dissolved in chloroform to obtain a stock solution of 10 mg/mL. Then, predetermined volumes of lipids were put into borosilicate glass probes in molar ratios of 95 mol% DMPC and 5 mol% DSPE-PEG 2000 to a final volume of 2 mL, which corresponds to 20 mg of lipids in total. Then, the organic phase was evaporated under nitrogen gas and the resultant thin lipid film was dissolved in tert-butanol under heating using a water bath. After complete dissolution, the tert-butanol solution of lipids was frozen in liquid nitrogen and subjected to freeze-drying under a vacuum overnight using a Savant Modulyo apparatus (Thermo Fisher Scientific, Waltham, MA, USA). The obtained lipid cake was hydrated with 1 mL of 150 mM sodium chloride at a predetermined temperature using a sonication bath. Hydration was performed until the lipid cake was fully hydrated and stopped immediately after. The obtained MLV liposome suspension was extruded using a 10 mL thermobarrel extruder through 100 nm Whatman Nuclepore polycarbonate filters. After 10 cycles of extrusion, the obtained large unilamellar vesicle (LUV) liposomes were stored in an Eppendorf tube for further assessment. For BAI encapsulation, the compound was dissolved in a mixture of chloroform and methanol in a volume ratio of 1:1, to obtain a 10 mg/mL stock solution, and added to the lipid solution in a predetermined amount. After mixing with lipids, BAI-loaded liposomes were obtained with the same procedure as used for empty LUV liposomes as described above.. The scheme for the preparation of liposomes with closed BAI is shown in Figure 1.

### 2.3. Liposome Determination of Mean Diameter Size, Polydispersity (PDI), and Zeta Potential of BAI-Loaded and Empty Liposomes

Size distribution, polydispersity index (PDI), and zeta potential values were measured using the Malvern NanoZS dynamic light scattering system (Malvern Instruments, Malvern, UK). Measurements were made in ultrapure MILLI-Q water at 25 °C. DLS measurement graphs were made using the built-in averaging software to acquire a single sample peak, made from the average of three separate runs (*n* = 3).

### 2.4. BAI Encapsulation Efficiency Determination and Process Optimization

To determine BAI encapsulation efficiency (EE%), the amount of BAI remaining in the liposomal bilayer after extrusion was measured. Since unencapsulated BAI precipitates on a polycarbonate filter, and only loaded BAI will pass through filters, encapsulated BAI could be measured photometrically at 325 nm, using a Shimadzu TCC-240A UV-Vis spectrophotometer (Shimadzu Corp., Kyoto, Japan). The EE% was calculated according to the following formula:

$$\mathrm{EE}\left(\%\right)=\frac{\mathrm{CaE}\left(\mathrm{mg}/\mathrm{mL}\right)}{\mathrm{CbE}\left(\mathrm{mg}/\mathrm{mL}\right)}\times 100\%$$

where CaE stands for BAI Concentration after Extrusion and CbE stands for BAI Concentration before Extrusion. The amount of BAI was calculated from the appropriate calibration curve and was in the range of 2.5–15 µg, R^2^ = 0.9999.

The encapsulation process was optimized to achieve a maximum EE% value by manipulating the temperature of film hydration and extrusion with changing ratios between lipids and the compound.

### 2.5. Long-Term Stability of BAI-Loaded Liposomes: Size, PDI, and Retention Evaluation

The size distribution and PDI for loaded BAI liposomes were measured immediately after preparation (day 0) and during storage, at 4 °C with 10 mM EDTA solution for 90 days, at predetermined intervals of 0, 1, 4, 8, 14, 21, 28, 60, and 90 days. Simultaneously, at the same time points, 100 µL of liposome suspension was transferred to an Eppendorf and then centrifuged at 13,000 RPM for 2 min. The supernatant, which was a suspension of liposomal BAI, was separated from the possible precipitate of crystals with which it was precipitated from the liposomes. The BAI concentration was determined as before and compared to the baseline BAI concentration on day 0, which was determined to be 100% of the encapsulated compound.

### 2.6. In Vitro Release of Baicalein from Liposomes in the Presence of Serum Albumins

To establish the interaction between BAI-loaded liposomes and serum proteins, the BAI-L sample was diluted to a final lipid concentration of 20 mg/mL. The liposomes were incubated with FBS (1:1, *v*/*v*) at 37 °C for 48 h in a Julabo TW12 water bath (Julabo GmbH, Seelbach, Germany). Samples (200 µL) at 0.083, 0.25, 0.5, 0.75, 1, 3, 8, 24, and 48 h were separated on Sepharose CL 4B minicolumns (5.5 × 70 mm). Separated samples were collected and the concentration of BAI and lipid were measured and compared to the initial concentration values before separation. Lipid concentration was established using an ammonium ferrothiocyanate assay [50] and BAI concentration was established as described previously.

### 2.7. Cryogenic Transmission Electron Microscopy (Cryo-EM) Visualization

Cryo-EM images were collected on a Tecnai G2 20 TWIN transmission electron microscope (FEI, Hillsboro, OR, USA) operating at 200 kV, whereas for the preparation of samples, an automated Vitrobot Mark IV (FEI, Hillsboro, OR, USA) was used. On a 200-mesh copper grid coated with perforated carbon film a negative glow discharging at 10 mA for 60 s, a 3 µL droplet of liposomes was applied. Then, after 3 s of blot time with a blot force of 15 in 90% humidity, the grid was plunge-frozen in liquid ethane cooled by liquid nitrogen. 

### 2.8. Cell Culture

AsPC-1 (from the ascites of a patient with PDAC) and BxPC-3 (primary pancreatic tumor) cells (ATCC, Manassas, VA, USA) were maintained with RPMI-1640 medium supplemented with 10% heat-inactivated fetal bovine serum (FBS), antibiotic-antimycotic mixture, and GlutaMAX solution under aseptic conditions in a Memmert ICO150 Med incubator (Memmert, Buchenbach, Germany). The NHDF (normal human dermal fibroblast) cell line was maintained with MEM-Alpha supplemented with 10% heat-inactivated fetal bovine serum (FBS), antibiotic-antimycotic mixture, and GlutaMAX as well. Cultures were maintained at 37 °C in a humidified atmosphere containing 5% CO_2_.

### 2.9. Cell Viability Assay Using MTT Colorimetric Test

The effect of the BAI-loaded liposomes on cell viability was determined using a quantitative colorimetric MTT assay adapted from Mosmann [51]. Cells were seeded in 96-well plates (4500 cells per well), in an appropriate complete cell culture medium, for 24 h. Cells were treated with BAI encapsulated in liposomes and BAI dissolved in DMSO in the range of 6.25–200 µM (an equivalent volume of DMSO was used as a negative control, maximal concentration was 0.5% *v*/*v*), as well as control unloaded liposomes, for 48 and 72 h. The medium containing the tested formulations was removed and the MTT solution (working solution: stock 0.5 mg/mL 10 times diluted in medium) was added to the wells, then the plates were incubated for a further 3 h. Subsequently, the MTT solution was replaced with DMSO (50 µL/well) to dissolve the purple formazan crystals. Absorbance was measured at 560 nm, with a reference wavelength of 670 nm, on an Asys UVM 340 Microplate Reader (Biochrom, Cambridge, UK). Results were expressed as the percentage of surviving cells, with respect to the control (the untreated cells), calculated as:

Cell Viability (%) = (AT/AC) × 100,

where AT is the absorbance of the treatment well (treated cells) and AC is the absorbance of the control well (untreated cells).

IC50 values were calculated using GraphPad Prism 7 for Windows (GraphPad Software, La Jolla, CA, USA).

### 2.10. Cellular Uptake

The cellular uptake of DiL liposomes by AsPC-1 and BxPC-3 cells was assessed by fluorescence microscopy. DiL was encapsulated into liposomes by dissolving DiL in ethanol to obtain 1 mg/mL stock solution. Then, Nile Red ethanol solution was added to the chloroform solution of lipids in a 1:999 (DiL: lipids) mass ratio. The organic phase was evaporated, and LUV-DiL liposomes were prepared with the same protocol as BAI or empty LUV liposomes. Cancer cells were seeded onto glass cover slides placed in 12-well culture plates. After 24 h incubation, the cell culture medium was replaced with a medium containing DiL-loaded liposomes. The cells were incubated at 37 °C for 5, 15, 30, and 60 min. Subsequently, cells were washed three times with PBS (37 °C), to remove excess liposomes, and fixed for 20 min in 4% paraformaldehyde, washed with phosphate-buffered saline (PBS), and stained with DAPI. Slides were analyzed using a Leica TCS SP8 confocal microscope (Leica-Microsystems, Mannheim, Germany) with an HC PL APO CS2 63×/1.40 oil objective. To excite DiL and 4′,6-diamidino-2-phenylindole (DAPI), a fluorescent probe that forms a complex by fixing to DNA, and 561 nm and 405 nm lasers (Leica-Microsystems, Mannheim, Germany) were used, respectively.

### 2.11. DNA Fragmentation Assay

The ability of baicalein to induce DNA breakage either alone or in the presence of copper ions was measured by determining the conversion level of the supercoiled form of plasmid DNA to open-circular and linear forms. The final, total reaction volume of 25 µL: 2 µL (1 µg) of pBR322 plasmid DNA (Thermo Scientific) in 10 mM Tris–HCl buffer (pH 7.5) was incubated with baicalein and/or 100 μM CuSO_4_ for 1 h at 37 °C. For control reactions, the plasmid DNA was incubated without the tested compound but in the presence of copper ions or DMSO (solvent control). The baicalein stock solution was prepared each time anew; the compound was suspended in DMSO and heated at 65 °C to dissolve before use. DNA fragmentation was analyzed by performing gel electrophoresis. After incubation, samples were mixed with 5 µL of gel loading buffer and loaded on 1% agarose gel. The gels were stained with SimplySafe (EURX) and photographed using a UV light in Azure 600 (Azure Biosystems, Dublin, CA, USA).

### 2.12. RealTime-Glo Apoptosis and Necrosis Assay

BxPC-3 cells were seeded in 96-well plates (10,000 cells/well) in the appropriate complete cell culture medium and adhered overnight. The next day, liposomal baicalein formulations (of 20 and 40 µM final concentrations) and freshly prepared RealTime-Glo Annexin V Apoptosis and Necrosis Assay reagent were added directly to the cell culture to detect apoptosis and necrosis. Cells were incubated in 5% CO_2_, at 37 °C, and in the meantime (after 2, 4, 6, 8, 24, and 48 h), luminescence and fluorescence were recorded by a GloMax Discover Microplate Reader (Promega, Madison, WI, USA). The reading parameters were luminescence: 0.5 s, integration time; fluorescence: excitation 475 nm, emission 500–550 nm. No-cell, no treated cells, and vehicle (empty liposomes) treated cell controls were prepared for background estimation, and untreated and carrier-treated response analysis, respectively. Results were expressed as the percentage of luminescence signal, with respect to the control (untreated cells), calculated as a percentage of signal (%) = (LT/LC) × 100, where LT is the luminescence of the treatment well (treated cells) and LC is the luminescence of the control (untreated cells). 

### 2.13. Preparation of Three-Dimensional Spheroids

To begin, 96-well plates were coated with 50 µL of 1.5% (*w*/*v*) sterile agarose dissolved in DMEM cell medium. After agarose cooling, BxPC-3 cells were seeded onto the prepared 96-well plates (15,000 cells per well) in filtered RPMI medium and immediately centrifuged in a Swing-Bucket rotor (3000 RPM, 20 min, 10 °C,) using Sigma 3–18K (Sigma-Aldrich, Saint Louis, MO, USA). Subsequently, plates were secured with parafilm and moved into a shaking incubator (135 RPM, 5% CO_2_, 37 °C) for 48 h. After removing from the shaking incubator, 50 µL of fresh RPMI medium was added and the plates were incubated for the next 48 h in the following conditions: 5% CO_2_, 37 °C. Afterward, spheroids were transferred to new plates; depending on the experiment, transparent (microscopy) or non-transparent, white, round-bottom PrimeSurface plates (Sumitomo Bakelite, Tokyo, Japan) were used for luminescence assay.

### 2.14. ATP Cell Viability Assay for 3D Spheroid Model

After baicalein treatment, cell viability was evaluated by measuring the adenosine triphosphate (ATP) levels using the Promega CellTiter-Glo 3D kit for spheroid cultures. ATP is an energy source of cells and its level correlates with cell number and viability. Reagents were prepared according to the manufacturer’s instructions.

Freshly obtained spheroids were transferred to a new opaque, white, round bottom PrimeSurface 96-well plate and treated with 120, 240, and 360 µM liposomal baicalein for 72 h. As a carrier control, 3D cultures were treated with non-loaded liposomes diluted the same way as liposomal baicalein. Control spheroids were not treated with liposomes, but an equal volume of medium was added (control untreated cells).

The viability of spheroids was determined according to the protocol of the manufacturer, Promega CellTiter-Glo 3D Assays. After the reagent adding plates were shaken for 10 min to allow spheroid/cell lysis, the luminescence signal was recorded by a GloMax Discover Microplate Reader.

### 2.15. Determination of Hemolysis of Human Erythrocytes

The evaluation of the hemolysis level was performed according to the procedure of Jaromin et al. [52]. The study protocol was approved by the Bioethics Commission at the Lower Silesian Medical Chamber (1/PNHAB/2018). BAI-L was added in a volume corresponding to a final concentration of 30 µM in the sample and further incubated (30 min, 37 °C) with freshly isolated human erythrocytes in PBS. The amount of released hemoglobin was detected at 540 nm after centrifugation. The appropriate controls were also prepared: negative (erythrocytes in PBS buffer) and positive (erythrocytes in distilled water).

### 2.16. Statistical Analysis

Data are presented as mean and standard deviation. Statistical analyses were performed using GraphPad Prism software (Version 7, GraphPad Software, San Diego, CA, USA) with a one-way ANOVA (Prism 7 for Windows) and Dunnett’s multiple comparisons test, with a 95% confidence interval.

## 3. Results

### 3.1. Preparation and Analysis of BAI-Loaded PEGylated Liposomes

**Figure 2:**
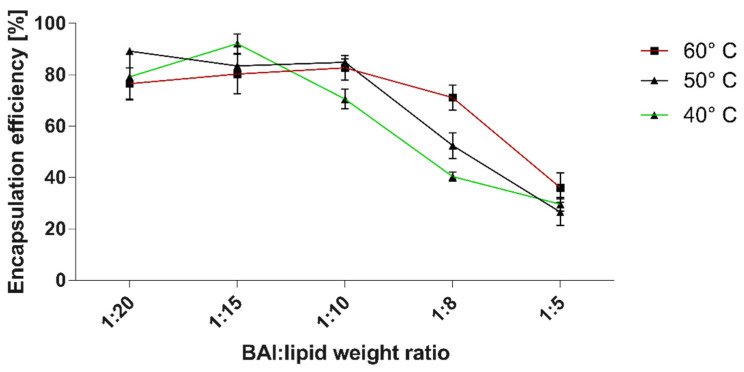
The efficiency of baicalein encapsulation into DMPC:DSPE-PEG2000 95:5 liposomes in correlation with the temperature of lipid-BAI “cake” hydration and lipid to BAI weight ratio. Data are the mean ± s.d, *n* = 3.

Due to the hydrophobic and non-water-soluble nature of BAI, the passive loading strategy was chosen for its encapsulation. For this preparation, the composition of DMPC and DSPE-PEG 2000 was used in the molar ratio of 95:5, with the additional step of lyophilization of the thin lipid-BAI film. We evaluated the encapsulation (EE%) efficiency at 40, 50, and 60 °C temperatures of hydration using 150 mM NaCl. The results of this assessment are presented in Figure 1. Maximal EE%, with the result of 92.1% ± 3.7, was achieved at 40 °C and for the BAI to lipid ratio of 1:15, with a minimal EE% value of 26.5 ± 5 at 50 °C and BAI to lipid ratio of 1:5. For every further experiment, BAI was loaded in a BAI:lipid ratio of 1:10 at 50 °C as a compromise between the drug:lipid ratio and loading. For this temperature, the EE% was 84.9% ± 1.3. In addition, in Figure 2, we have shown a scheme for preparing the encapsulation of baicalein in liposomes.

### 3.2. Characterization of Obtained BAI-Loaded Liposomal Suspension by Dynamic Light Scattering (DLS) System

**Figure 3:**
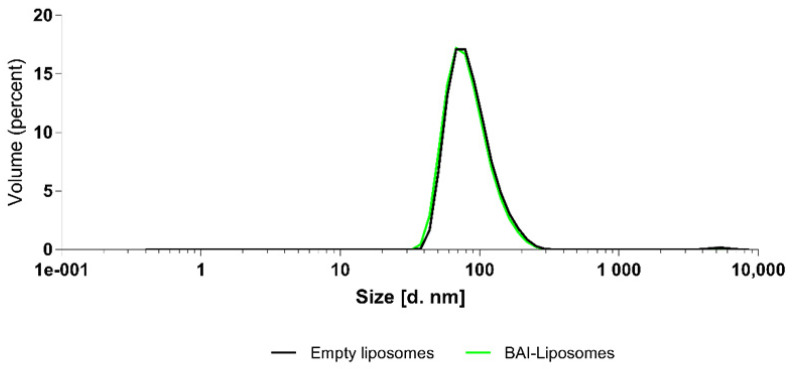
Size distribution of BAI-loaded and empty, unloaded, liposomes.

**Figure 4:**
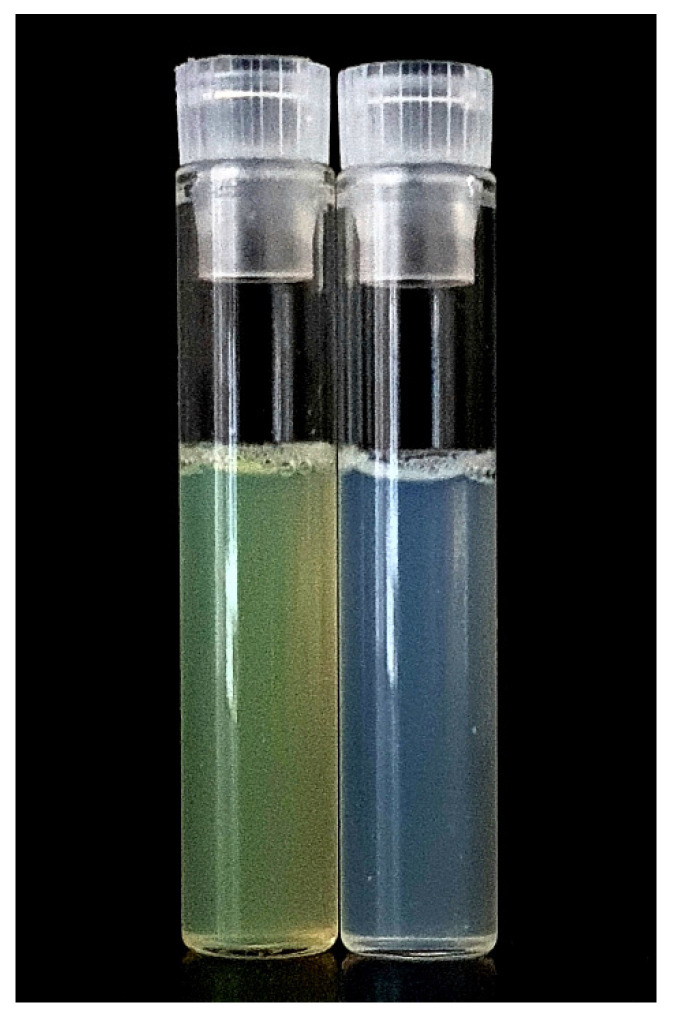
The visual appearance of the obtained liposomal suspensions: BAI-loaded (**left**) and empty, non-loaded, liposomes (**right**).

We evaluated the obtained BAI liposomal suspension in terms of the size, homogeneity, and zeta potential parameters using the Malvern NanoZS Dynamic Light Scattering (DLS) system. Figure 3 shows the size distribution of the BAI-loaded liposomes and Table 1 shows the results of these measurements for loaded BAI liposomes as empty liposomes. The obtained BAI-loaded liposomal suspension is characterized by a mean size of 100.9 ± 2.7 nm, PDI of 0.124 ± 0.02, and zeta potential of 9.84 ± 0.4 mV, which meets the criteria for a nanoformulation suitable for potential intravenous administration. Figure 4 shows a photograph of the obtained BAI-loaded liposomes in comparison to a non-loaded suspension. The visual appearance of the BAI liposomes in the solution was green-colored, highly translucent, and opalescent. These empty liposomes were very similar in appearance but without green color. The color of the liposome suspension with encapsulated BAI is a result of the color of the compound itself, which is green-yellow, and the slight bluish hue of the liposome suspension. Empty liposomes lack this coloration, as they do not have baicalein encapsulated in their phospholipid bilayer.

### 3.3. Cryogenic Transmission Electron Microscopy (Cryo-TEM) Visualization of Nanoparticles

**Figure 5:**
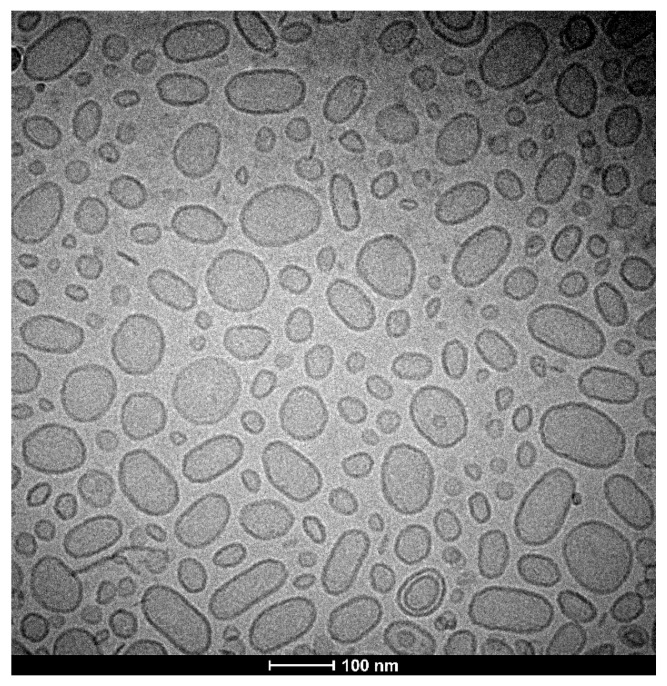
Cryo-TEM image of a BAI-loaded liposomal suspension.

For microscopic visualization, the cryo-TEM technique was used. We visualized BAI-loaded liposomes, and the results of this cryo-TEM imaging are presented in Figure 5. The size range agrees well with the DLS measurements and the liposomes are almost entirely unilamellar.

### 3.4. Long-Term Stability Studies of BAI Liposomes

**Figure 6:**
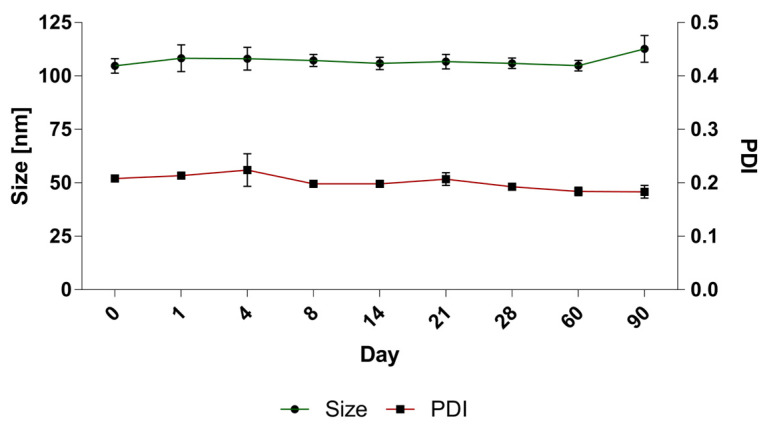
The stability of BAI-loaded liposomes: changes in size and PDI values. Data are the mean ± s.d, *n* = 3.

**Figure 7:**
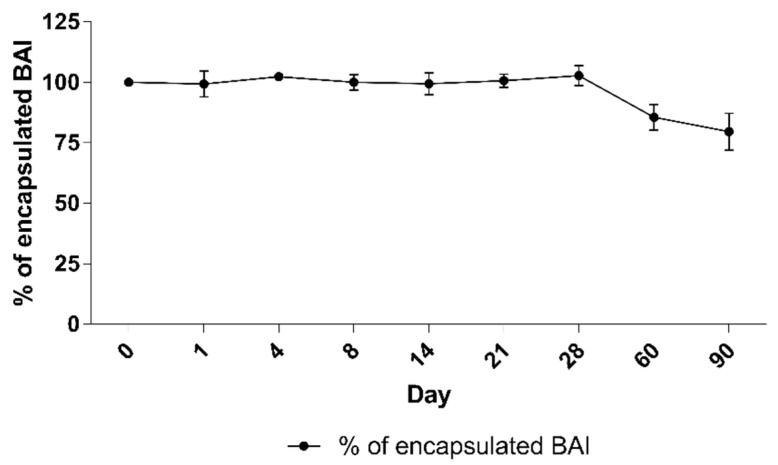
The stability of BAI loaded liposomes: retention of BAI from liposomes during 90 days of incubation. Data are the mean ± s.d, *n* = 3.

A long-term stability assay was conducted for BAI liposomes. For size and PDI evaluation purposes, the liposomes were incubated in the presence of 10 mM EDTA, for 90 days at 4 °C, and the size and homogeneity, which is represented by the PDI parameter, were monitored. In order to determine the leakage of the compound from the liposomes, 100 uL of liposomes were taken from the incubated sample on each measurement day and centrifuged at 13,000 RPM for 2 min. The supernatant, which is a suspension of liposomal BAI, was then separated from the precipitate-crystallized BAI that leaked from the liposomes, and the concentration of the remaining encapsulated BAI in the liposomes was measured analogously to Section 3.1. The sample quality was maintained during the whole 90 days of incubation. The initial value of the size at day 0 was 104.7 nm ± 3.4 and 112.6 ± 6.2 at day 90, after the end of the experiment. The PDI remained low, indicating that the initial homogeneity was retained: from 0.2 ± 0.01 at day 0 and 0.18 ± 0.01 at day 90. Detailed results are presented in Figure 6. When measuring the retention of the compound in liposomes, visible crystallization was observed only after 60 days of incubation of the samples. Ultimately, a loss of about 20% of the encapsulated BAI in the liposomes was observed, and on the day the experiment ended, the percentage of encapsulated BAI was 79.6% ± 7.6 The exact results of this experiment are shown in Figure 7.

### 3.5. BAI-Liposome Stability in Presence of Serum Albumins and Hemolytic Activity

**Figure 8:**
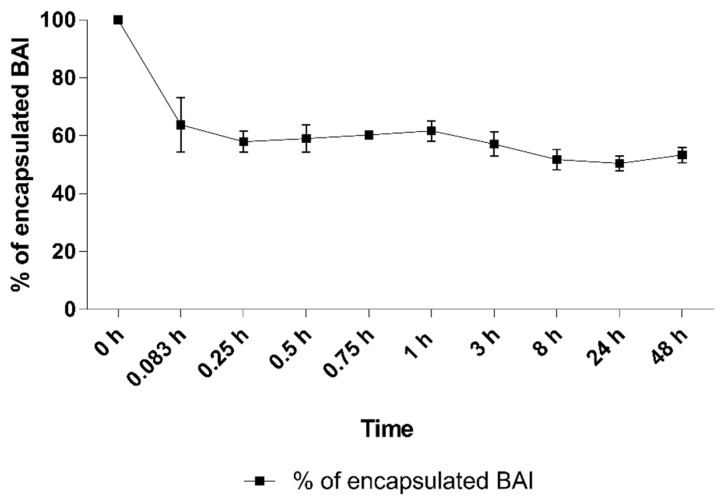
The retention of BAI in liposomes during 48 h of incubation in 50% FBS. Data are the mean ± s.d, *n* = 3.

One of the crucial aspects of nanoformulations is their stability after intravenous administration and their interaction with serum proteins afterward. We experimented by incubating the BAI-loaded liposomal suspension in the presence of 50% FBS for 48 h at 37 °C. The results are presented in Figure 8. An initial drop to 63.8% ± 9.3 was observed after the first 5 min of incubation. However, this value did not change significantly during the rest of the incubation, and at the end of the experiment, 53.4% ± 2.7 of the BAI was still encapsulated in the liposomes. In addition, the stability of the preparation in terms of its size and homogeneity was measured. Based on the results in Table 2, there was no phenomenon of aggregation of liposomes in contact with FBS, which would manifest itself in significant changes in the size and homogeneity of liposomes. 

When considering the potential intravenous administration of the developed formulation, we also assessed its potential hemolytic activity. It was found that BAI-L causes 0.99% hemolysis at a BAI concentration of 30 µM, which is a negligible value.

### 3.6. Cytotoxic Activity of BAI Liposomes towards PDAC Cell Lines and Normal NHDF Cell Line

**Figure 9:**
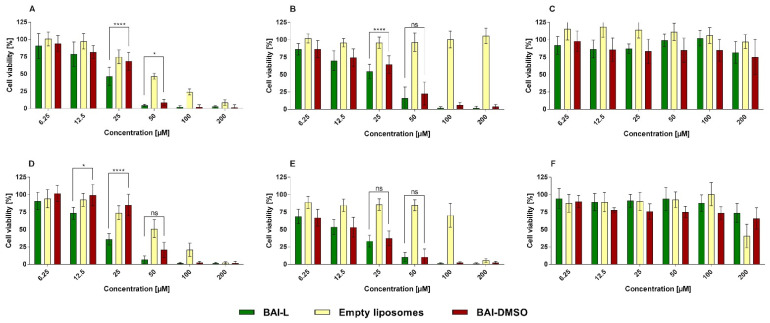
The cytotoxic effect of baicalein loaded into DMPC:DSPE-PEG2000 95:5 liposomes, or in the free form in DMSO, as determined by MTT assay, after 48 (panels (**A**–**C**)) and 72 h (panels (**D**–**F**)) of incubation for AsPC-1 (panel (**A**,**D**)), BxPC-3 (panel (**B**,**E**), and NHDF (panel (**C**,**F**)) cell lines. Non-loaded liposomes were tested as well. For a dose of 12.5, 25, and 50 µM, the statistical significance of differences between free and loaded BAI, evaluated by GraphPad Prism 7, is indicated by asterisks (0.1234 Ns, 0.0332 *, >0.0001 ****), with 95% confidence. ns stands for “non-significant”. Data are the mean ± s.d, *n* = 12.

To evaluate the anticancer potential, and suspected specificity of BAI towards cancer cells, of the BAI and BAI liposomes, we investigated their in vitro cytotoxicity against two human pancreatic cancer cell lines (AsPC-1 and BxPC-3). Liposomes were also tested on a normal human dermal fibroblast cell line (NHDF). During experiments, cells were incubated for 48 and 72 h with BAI-L in a DMSO solution (free compound) and BAI-loaded and unloaded liposomes. The experimental outcome was established using the MTT test, which is based on the detection of the oxidoreductive enzymes (especially succinate dehydrogenase) in the mitochondria of living, fully metabolizing cells. During the experiment, cells were incubated with a range of concentrations (6.25–200 µM) of BAI dissolved in DMSO (which is commonly used as a solvent for drug testing), which was treated as a positive control, or BAI loaded into PEGylated liposomes. As negative controls, non-loaded liposomes were used. DMSO as another negative control was tested as well. The results are presented in Figure 9, whereas the calculated IC50 values are presented in Table 3.

### 3.7. Baicalein Formulation Induces Apoptosis in Pancreatic Cancer BxPC-3 Cell Line

**Figure 10:**
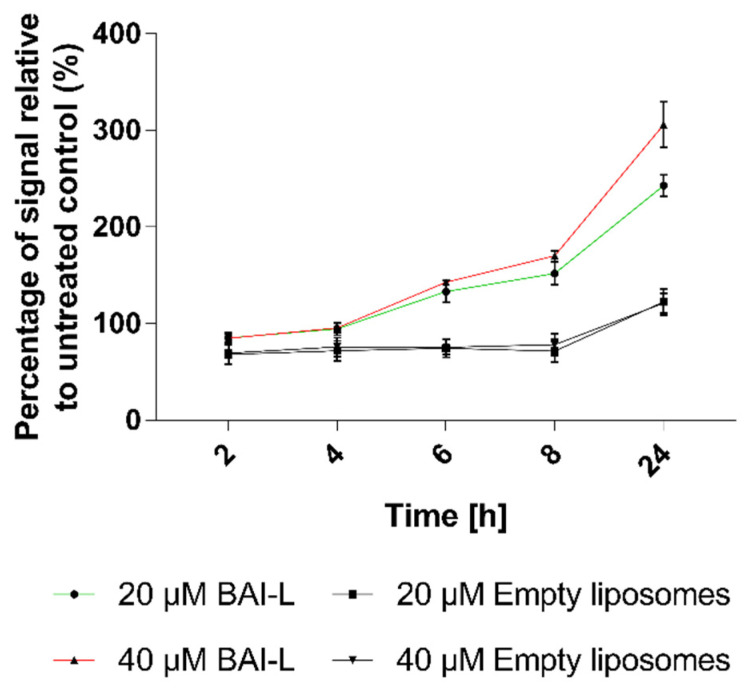
The apoptotic effects of liposomal baicalein (BAI-L) on the human pancreatic cancer cell line BxPC3 as determined by the RealTime-Glo™ Apoptosis assay. Data are the mean ± s.d, *n* = 10.

To determine whether observed cell death was mediated via apoptosis or necrosis, we used the RealTime-Glo Annexin V Apoptosis and Necrosis Assay. This test is a live-cell, real-time kinetic assay which measures phosphatidylserine (PS) molecules’ exposure on the outer leaflet of the cell plasma membrane as a signal for apoptosis (luminescence), and loss of membrane integrity as a signal for necrosis (fluorescence). In this experiment, we tested the BxPC-3 line as it is more sensitive to the formulation. The results presented in Figure 10 show a significant dose-dependent increase in Annexin-V luminescence at the time of the experiment, indicating the occurrence of apoptosis. The apoptosis signal was observed 6 h after the addition of the formulation and it increased over time (it was higher for the higher baicalein concentration). In the case of non-loaded liposomes, an apoptosis signal was also observed, but only after 24 h, and it was considerably lower than the signal observed for loaded liposomes. In the examined time range, there were no changes in fluorescence, thus no necrosis was detected.

### 3.8. Cellular Uptake of BAI-Loaded Liposomes

**Figure 11:**
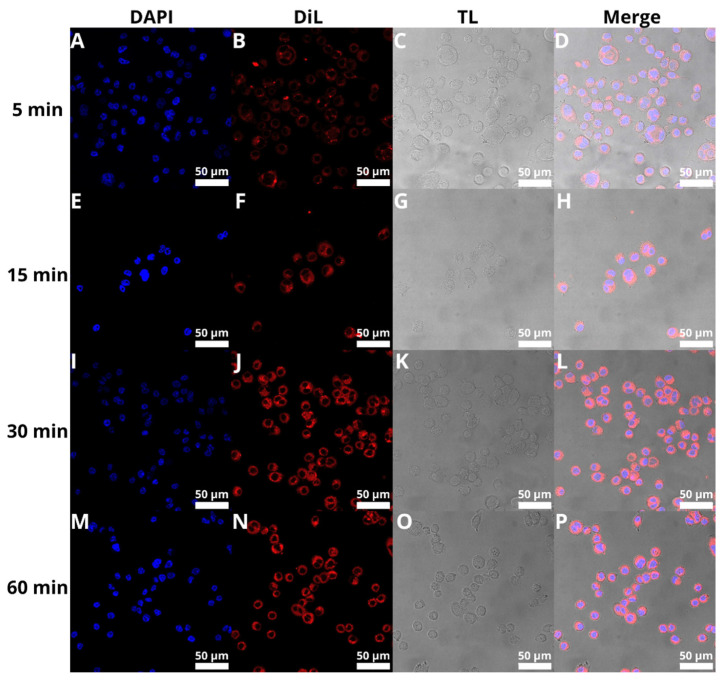
Visualization of the cellular uptake of DiL-loaded BAI-liposomes by the AsPC-1 pancreatic cell line during 5 min (**A**–**D**), 15 min (**E**–**H**), 30 min (**I**–**L**), and 60 min (**M**–**P**) of incubation. Scale bar = 50 µM. DAPI stands for fluorescence signal of DAPI stain, DiL stands for fluoresce signal of DiL stain, TL stands for transmitted light, Merge stands for merged signal from three separate channels.

**Figure 12:**
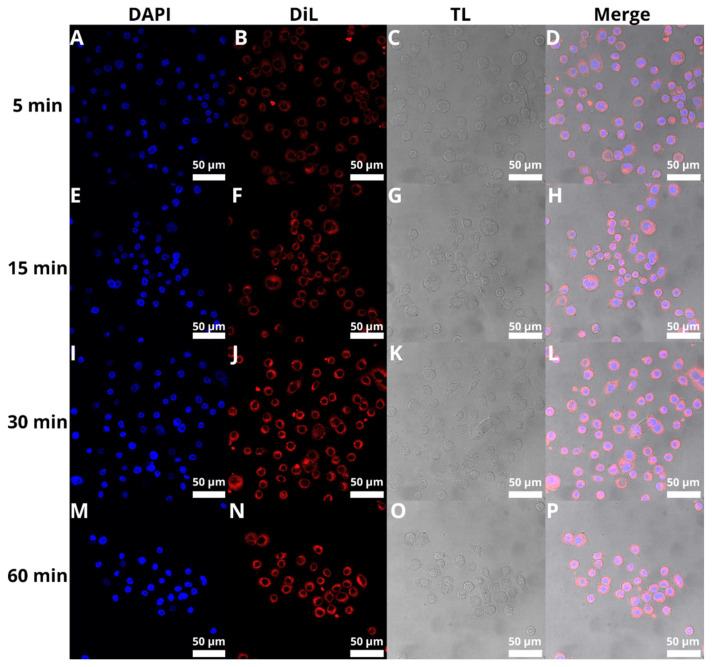
Visualization of the cellular uptake of DiL-loaded BAI-liposomes by the BxPC-3 pancreatic cell line during 5 min (**A**–**D**), 15 min (**E**–**H**), 30 min (**I**–**L**), and 60 min (**M**–**P**) of incubation. Scale bar = 50 µM. DAPI stands for fluorescence signal of DAPI stain, DiL stands for fluoresce signal of DiL stain, TL stands for transmitted light, Merge stands for merged signal from three separate channels.

The next step was to evaluate the cellular uptake of the liposomes. For this purpose, we labeled liposomes with Nile Red, which is commonly used for bioimaging studies [53,54,55]. Confocal microscopy observation was performed using fluorescence signals from two fluorophores: one from cell nuclei stained with DAPI, the second from DiL encapsulated in liposomes, with the addition of transmitted light as well. We performed this assay for 5, 15, 30, and 60 min. The results, presented in Figure 11 and Figure 12, show that the liposomes were effectively internalized within AsPC-1 and BxPC-3 cells after this incubation period, with full internalization after 30 min of incubation.

### 3.9. Three-Dimensional Cultures

**Figure 13:**
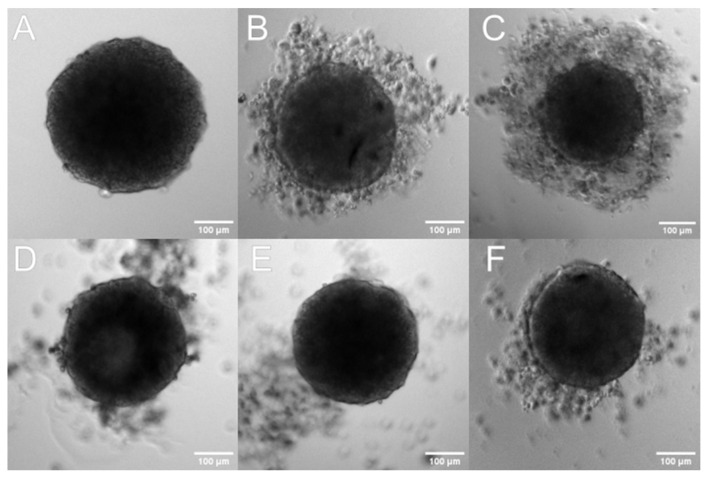
The effect of liposomal baicalein on the shape and viability of spheroids. Three-dimensional spheroids were generated in agarose-coated wells with subsequent centrifugation. The generated spheroids were treated for 72 h with BAI-L 1—Representative microscopy images of spheroids: (**A**) spheroid before treatment (time 0), (**B**–**F**) spheroids treated with: (**B**) liposomal baicalein (120 µM), (**C**) liposomal baicalein (360 µM), (**D**) RPMI, and (**E**,**F**) non-loaded liposomes diluted with a baicalein formulation of 120 µM and 360 µM, respectively. Scale bar = 100 µm.

In addition to traditional two-dimensional (2D) cultures, we also used three-dimensional cultures (3D; spheroids). The 3D system is believed to reflect more accurately the tumor-like microenvironment, especially for solid tumors such as pancreatic ductal adenocarcinomas characterized by profuse desmoplasia. A growing number of studies have suggested that the 3D model is physiologically a more relevant model which provides more accurate cytotoxicity information for the initial drug screening and could be a bridge between the classic 2D culture and animal experiments [56]. For 3D model analysis, the BxPC-3 line was selected because it was more sensitive to the tested formulation. To obtain multicellular tumor spheroids BxPC-3 cells were seeded on non-adhesive surfaces to avoid cell attachment. Additionally, cells were harvested and grown for two days in a shaking incubator and then transferred to a classical incubator for two days. As a result, the cells formed dense, self-assembled clusters—spheroids. Using this method, we were able to obtain a high number of reproducible spheroids that were uniform in shape. In cytotoxicity tests, the 4-day-old spheroids were exposed for 72 h to different concentrations of tested liposomal formulation, then their condition was analyzed by light microscopy and bioluminescence quantitative measurements of adenosine-5’-triphosphate (ATP) (CellTiter-Glo 3D Cell Viability Assay). Microscope imaging was used to assess the morphological changes, size distribution, and spheroids’ shape analysis. As can be seen from Figure 13, in the presence of the tested formulation, spheroids were generally less dense and a thick halo of loose cells appears, which is correlated with baicalein concentration, and the core has a more irregular shape (the dense core was smaller and had less defined boundaries) (Figure 13B,C). Spheroids treated with empty liposomes and untreated control spheroids revealed a typical architecture of a dense spheroid containing a necrotic core surrounded by a few free cells.

**Figure 14:**
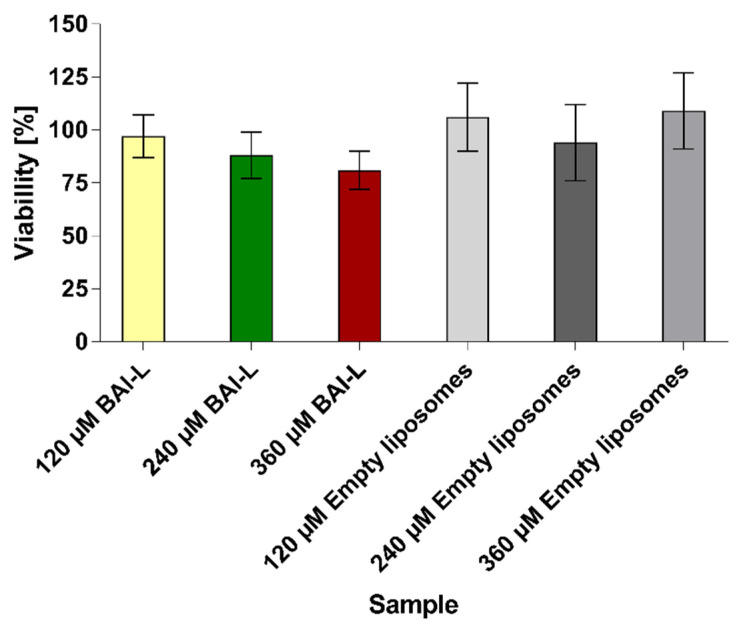
Cell viability was calculated using the CellTiter-Glo 3D Cell Viability Assay for 3D spheroids treated with liposomal baicalein (120, 240, and 360 µM) and non-loaded liposomes (diluted the same as loaded formulations). Data are the mean ± s.d, *n* = 3.

The number of viable cells in the spheroid cultures was estimated based on the quantitation of the ATP, which was used as an indicator for the presence of metabolically active cells. We evaluated the effects of baicalein formulation with the CellTiter-Glo 3D luminescent assay, where ATP presence generates a luminescent readout. For the 120 µM, 240 µM, and 360 µM BAI-L concentrations, the cell viability measured by the ATP level was 97% ± 10, 88% ± 11, and 81% ± 9, respectively. For the non-loaded liposomes diluted in the same way as the baicalein formulation, the estimated viability was 109% ± 18 (Figure 14). The presented data were calculated from three independent experiments. In each experiment, a minimum of 10 spheroids was tested per formulation concentration.

### 3.10. DNA Fragmentation Induction by BAI

**Figure 15:**
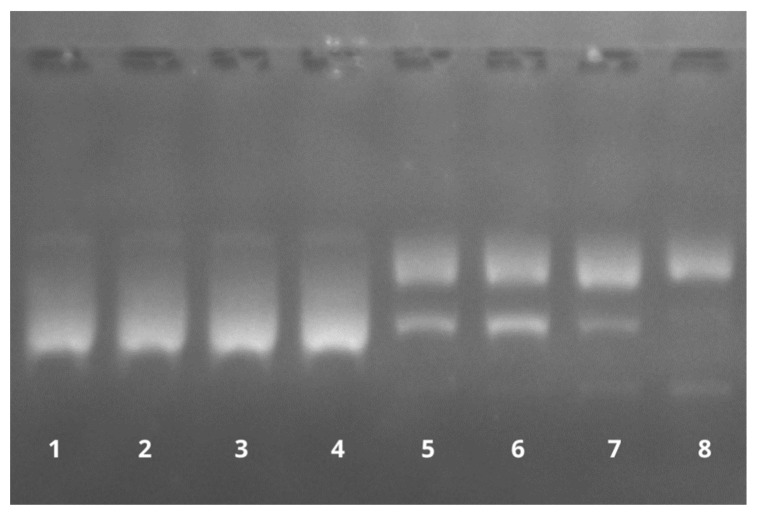
The effect of baicalein on plasmid pBR322 DNA cleavage in the presence of Cu(II). Lane 1: control (plasmid DNA without any addition); Lane 2: DNA with Cu(II); Lane 3: DNA with 100 µM baicalein; Lane 4: DNA with Cu(II) and DMSO; and Lanes 5–8: DNA with Cu(II) and baicalein (25, 50, 100, and 150 µM, respectively).

As seen in Figure 15, the BAI is able to induce DNA cleavage in the presence of copper ions. DNA strand breaks caused the conversion of the supercoiled DNA into nicked and linear forms, and further, with higher baicalein concentrations, smaller fragments were also visible (lanes 5–8). Notably, for Cu(II) or baicalein alone, DNA damage was not detected (lanes 2 and 3), with results that were the same as for the solvent (DMSO) in the presence of Cu(II) in the reaction mixture (lane 4). These results suggest the participation of both baicalein and Cu(II) in the observed DNA damage.

## 4. Discussion

Encapsulation of hydrophobic compounds is a common strategy to enhance or even regain their bioactivity and bioavailability. A wide range of flavonoids and polyphenols are reported as potentially valuable and interesting anticancer agents [57]; however, due to their water insolubility or structural instability in biological fluids, this potential is marginalized and highly reduced [58]. That is why liposomes have emerged as a suitable solution to this issue. In the case of lipophilic drugs, they can be treated as a substitute for solvent, increasing the tolerance by the organism, unlike solvents such as DMSO or methanol which can be used in molecular in vitro analysis but not in intravenous administration. However, liposomes are still a rather complex system with a number of variables, not least because of the rather complicated process of producing liposomal drugs. Therefore, any potential new therapy will require a huge investment of time in both transferring the technology from the micro to the macro scale and optimizing such large-scale production.

As we have mentioned, BAI is reported as a potentially excellent weapon against PDAC. However, this potential is strictly limited by its very low water solubility (0.054 mg/mL) [59], which means there is no possibility to achieve a significant biological impact after administration. There have already been reports of BAI encapsulation into liposomes, but none of them were tested against pancreatic cancer cells or tissues. Wang et al. tested a BAI-liposomal suspension against K562 myeloid leukemia cells and reported a liposomal suspension with the desired size of 100, 200, or 400 nm, with PDI values around 0.1 and a zeta potential around -31 mV. However, this group did not provide detailed size distribution graphs or PDI distribution values. Unfortunately, there are no data from this work about the EE% of BAI [60]. Another BAI loading procedure was reported by Fang et al. [61]. They obtained a BAI-loaded liposomal suspension with a size ranging from 135.7 ± 9 nm to 154.2 ± 2 nm, which could be suitable for potential intravenous administration. However, this was correlated with a relatively low entrapment efficiency (below 35%) and final BAI concentrations (below 5 µg/mL) as well as with a high PDI value ranging from 0.462 to 0.503, indicating the relatively high heterogeneity of the sample. Another trial was reported by Liang et al. [62]. They encapsulated BAI with an EE% of 41.5 ± 4.7, but once again this formulation was characterized by a too-high PDI value (0.43) and the liposomes were too large (~709 nm) to be qualified for intravenous administration. Compared to these results, our developed formulation is superior in terms of size control (almost exactly 100 nm), homogeneity, characterized by a PDI value of 0.124 ± 0.02, and an entrapment efficiency of 84.9% ± 1.3. Importantly, we were able to produce formulations with a high final concentration of BAI in the sample, where 2 or 5 mg/mL BAI was easily achievable. For the best BAI to lipid ratio for the encapsulation, we chose a 1:10 BAI:lipid ratio and a 50 °C hydration temperature. This procedure resulted in very repeatable BAI encapsulations with relatively small differences in EE% between preparation series. It should be noted, however, that the DMPC-based BAI-loaded formulations obtained by us are characterized by a slightly positive charge. We use the phrase “slightly” in this context because, in the case of typically cationic formulations based on DOTAP, DDAB, or SA, the zeta value of such formulations is about +50 mV (data not shown). However, based on the many measurements made of the same formulation at different time points while maintaining good sample size, homogeneity, and retention parameters, we believe that the slight cationic potential of this formulation will not be a significant problem.

The liposomes prepared in this study with the DMPC-based formulation of BAI showed high stability during long-term storage. The size and homogeneity did not change significantly during the 90-day storage period. Samples did not aggregate, collapse, or precipitate, which could have manifested in a significant change in the PDI value. During the short-term stability assay in 50% FBS, the rapid loss of the payload was observed during the first 5 min of incubation. Almost 40% of the BAI leaked from the liposomal bilayer and bound to the serum albumins. However, this significant loss of payload was only noticeable once during the first 5 min of the experiment. The size and homogeneity of the samples during this assay did not change significantly, indicating a lack of aggregation or precipitation. Based on these results, it can be concluded that the sample reacts in a limited way with proteins, only in terms of leakage of the compound from the carrier, without the phenomenon of aggregation of the sample. It is also worth noting that the formulation has a high stability in the context of compound leakage from liposomes. The formulation showed no signs of crystallization of the compound from the carrier for the first month of measurements up to the 90th day of measurement, where it reached a value of 79.6% ± 7.6. The absence of signs of retention of the compound during the first 28 days of incubation, while maintaining the parameters of size and polydispersity, testifies to the high quality of the obtained formulation and will manifest in the ability to safely store the formulation as well as work with it without problems.

The liposomes were devoid of harmful activity toward human erythrocytes. The estimated level of hemolysis was very low, which confirms that it is a completely safe formulation that could potentially be used for intravenous administration. After obtaining a very promising, stable, and homogeneous sample with a very high encapsulation efficiency, we conducted cytotoxic tests of the formulation we developed. We used two times, 48 and 72 h, for the conducted test. We demonstrated the high potential of the obtained formulation against two pancreatic cancer cell lines: AsPC-1 and BxPC-3. Importantly, the formulation significantly showed no toxicity against the normal NHDF cell line in the tested concentration range. Thus, we can confirm its potential specificity as well as the reduced toxicity of potential therapy based on such BAI liposomes. However, the toxicity of the empty carrier remains in question, especially against the metastatic AsPC-1 cell line. Does the effectiveness of the formulation actually come from the encapsulated baicalein, or is the carrier itself toxic? At this point, we are not able to clearly answer the question of where this toxicity of the empty carrier comes from. A working hypothesis is that the phenomenon may be closely related to the composition of the liposomes. At room temperature, the bilayer of such liposomes is in an ordered, rigid state, understood as a “gel phase”. The thermotropic phase transition of such a formulation constructed from DMPC and DSPE-PEG2000 is about 28 °C. Under cell culture conditions, the temperature is 37 °C. When it is administered in large quantities to a two-dimensional cell culture, the liposome membranes are most likely to fuse with mitochondrial membranes, followed by liquefaction and the consequent release of cytochrome C and cytochrome-dependent cell death [63,64]. The use of carriers with a stiffer bilayer composed of lipids such as DPPC (Dipalmitoylphosphatidylcholine) and HSPC (Hydrogenated soy phosphatidylocholine) would certainly help solve this problem, while during our experiments, the use of such liposomes drastically reduced the efficiency of BAI encapsulation, or even prevented it. Liposomes constructed from DMPC had the best and most reproducible ability to encapsulate this flavonoid, so the decision was made to use it. However, this phenomenon does not disqualify our liposomal baicalein as a potentially valuable formulation against PDAC. First of all, it is important to note the differences in cytotoxicity between the empty carrier and the BAI-loaded liposomes at 25 and 50 µM concentrations, which clearly show the cytotoxic effect of only BAI encapsulated in liposomes. Second, this effect is particularly apparent for the metastatic line, where the primary BxPC-3 line shows sensitivity to the empty carrier at a much higher concentration range while maintaining the same sensitivity to liposomal BAI. Furthermore, based on our RealTime-Glo Annexin V Apoptosis and Necrosis Assay, we established the apoptosis-related cell death pathway. However, empty vessels also cause apoptosis of the cells, but this could also be related to the toxicity of the reagent or the “harsh” conditions of the experiment. Still, the difference between the loaded and unloaded liposomes is clearly visible, especially for the higher concentration of BAI-L used, where results from empty carriers are basically the same for both amounts.

However, we felt that a basic two-dimensional cell model would be insufficient to verify the true potential of our formulation and felt the need to go a level higher and verify the activity of our liposomal baicalein on a more complex system. That is why, for further verification, we conducted an activity test of our formulation on a 3D cell culture model in the form of a spheroid. The spheroid model is a more complicated cell culture system that better reflects tumor phenomena, such as hypoxia and the formation of non-cellular structures such as the extracellular matrix, and allows us to study the ability of the carrier to penetrate deep into a 3D cell mass, such as a spheroid [65,66]. Thanks to the spheroid culture, it is possible to verify the effectiveness of a given therapy in case of encountering such problems as a physicochemical barrier in the form of charged and dense hyaluronans, which are key components of the extracellular matrix, or a completely different cellular response from units that have their metabolism “switched” to “live as one”. The spheroids we prepared were characterized by a spherical, three-dimensional shape and a size of about 350 µm. We visualized control spheroids, after administration of the medium alone, and spheroids after administration of 120 and 360 µM BAI-L doses. We evaluated the cytotoxic potential of BAI-loaded and unloaded liposomes in three concentrations: 120, 240, and 360 µM. Based on our measurements, for a single BAI-L dosage, we did not observe any significant effect for the 120 µM concentration, whereas 240 µM reduced the viability of the cells to 88% and 360 µM to 81%. This visible decrease in BAI-L cytotoxicity could lead to two conclusions: first, three-dimensional cell structures are drastically more resistant to treatment compared to two-dimensional, “classic” cell cultures. As mentioned, this could be related to a completely different cell response due to the more complicated system. Secondly, in any analysis of potential therapeutic sample cytotoxicity, more time should be devoted to studying systems that better reflect the actual conditions of the tumor, such as the spheroid model, which at least reflects the need to overcome the compacted mass of cells [67,68]. However, it is important to pay attention to the results obtained from the measurements and contrast them with the microscopic images. Microscopic analysis of a concentration of 120 µM may indicate that a partial biological effect has been obtained for BAI-L, whereas 360 µM shows severe damage to the spheroid structure. However, analysis with the CellTiter-Glo 3D luminescent assay shows different, one might say, less spectacular, results. Therefore, the interpretation of results obtained from more complex 3D models should be approached with caution, even more so if complete destruction of the spheroid structure is not observed. The last noteworthy conclusion is that even when using much higher concentrations of empty liposomes than on the 2D model, they possess no negative effect on the spheroids.

Polyphenols are well known for their antioxidant properties, but it has been previously reported that these compounds under certain conditions (such as in the presence of transitional metal ions, e.g., copper) might also act as prooxidants, catalyzing DNA degradation. According to the literature data, the DNA degradation ability depends on the localization of hydroxyl groups at different positions of the benzene ring [69]. The described prooxidant action may be an important mechanism for BAI anticancer and cytotoxic properties, so we decided to test the ability of BAI to induce DNA breakage. Of note, the DNA degradation caused by this flavonoid occurs only in the presence of Cu^2+^ ions. The ability of flavonoids to bind to metal ions and form complexes has already been documented [70,71,72]. Perhaps this phenomenon is one possible reason for the anti-tumor activity of BAI. We should also note that such a complex is definitely much more specific against cancer cells than NHDF control cells. Perhaps this could be explained by a higher concentration of copper ions inside cancer cells compared to normal cells, or is the immune mechanism of healthy cells independent of copper ions at this point? It has been proven that the overall concentration of copper ions is higher in cancerous tissues compared to non-cancerous tissues [73,74] Some reports of increased copper ion levels and impaired copper/zinc metabolism in pancreatic cancer are available [75], while no more detailed data on this topic have been published to date. We believe that it is necessary to pay more attention to the reactions between flavonoids and transition metal ions, such as copper or iron, which, firstly, can react with flavonoids to form potentially new and interesting compounds and, secondly, can simultaneously support their anticancer effects through free radical reactions based on the Fenton reaction [76,77].

Confirmation of BAI-L’s “from within” effect can also be provided by the test of liposome internalization into cells. There is clearly full internalization of the carrier after 30 min of incubation, with no difference in signal intensity after the full 60 min of the experiment. This demonstrates that the liposomes are efficiently transported into the cells, where the cargo in the form of BAI is released and can effectively destroy the cancer cell. These results of liposome internalization can be compared with other experiments where rapid liposome uptake was also reported [78,79,80,81].

## 5. Conclusions

The liposome form of BAI has an appropriate size and homogeneity, as well as stability, and is non-hemolytic. Further development of this formulation requires subjecting the formulation to more organized structures, such as spheroids or higher organoids, and consequently conducting a study on a mouse model for the actual efficacy of liposomal baicalein. This is the first step towards the use of baicalein against pancreatic cancer, and we hope it is a step in the right direction. By obtaining a liposomal formulation of high quality and efficacy, BAI qualifies for potential use as monotherapy or as adjunctive therapy to treatment with gemcitabine or other available liposome-based therapies.

## Figures and Tables

**Table 1:** The characterization of liposomes: size, PDI, and zeta potential values. Data are the mean ± s.d, *n* = 3.

Sample	BAI-L	Empty Liposomes
Size (nm)	100.9 ± 2.7	99.6 ± 1.1
PDI	0.12 ± 0.02	0.14 ± 0.01
Zeta potential (mV)	9.8 ± 0.4	7.8 ± 0.3

**Table 2:** The changes in the size and homogeneity (PDI) values during 48 h of the FBS stability assay. Data are the mean ± s.d, *n* = 3.

Time (h)	0	24	48
**Size (nm)**	94.1 ± 1.3	93.3 ± 0.3	103.9 ± 2.9
**PDI**	0.13 ± 0.01	0.07 ± 0.01	0.15 ± 0.02

**Table 3:** The calculated IC_50_ values(µM) for AsPC-1, BxPC-3, and NHDF cell lines. Non-toxicity was determined to be in the concentration range of 6.25 to 200 µM. Data are the mean ± s.d, *n* = 12.

	Cell Line					
	AsPC-1		BxPC-3		NHDF	
Sample	IC50 48 h	IC50 72 h	IC50 48 h	IC50 72 h	IC50 48 h	IC50 72 h
**BAI-L**	24.9 ± 4.2	21 ± 3.6	27.6 ± 4.1	23.1 ± 4.3	Non-toxic	Non-toxic
**Empty liposomes**	41.7 ± 1	51.9 ± 7	59.4 ± 34	112.3 ± 7.9	Non-toxic	140 ± 4.6
**BAI-DMSO**	30.4 ± 1.6	36 ± 3.6	34 ± 6.7	25.1 ± 7	Non-toxic	49.3 ± 12
**DMSO**	Non-toxic	Non-toxic	Non-toxic	Non-toxic	Non-toxic	Non-toxic

## Data Availability

The data are stored by the corresponding author and are available upon request.
